# P-1895. Outpatient Parenteral Antimicrobial Therapy Multidisciplinary Partnership Reduces Readmissions and Emergency Center Visits in a Comprehensive Cancer Center

**DOI:** 10.1093/ofid/ofae631.2056

**Published:** 2025-01-29

**Authors:** Natalie J Dailey Garnes, Meagan Rowan, Jaime Pena, Meghan Kamath, Ashlyn Anastasia Florack, Janice Finder, Jessica E Baulis, Wesley Amistad, Ying Jiang, Javier Adachi

**Affiliations:** University of Texas MD Anderson Cancer Center, Houston, Texas; University of Texas MD Anderson Cancer Center, Houston, Texas; MD Anderson Cancer Center, Houston, Texas; The University of Texas MD Anderson Cancer Center, Houston, Texas; The University of Texas MD Anderson Cancer Center, Houston, Texas; University of Texas MDAnderson, Houston, Texas; UT MD Anderson, Houston, Texas; University Of Texas MD Anderson Cancer Center, Houston, Texas; The University of Texas MD Anderson Cancer Center, Houston, Texas; MD Anderson Cancer Center, Houston, Texas

## Abstract

**Background:**

Outpatient parenteral antimicrobial therapy (OPAT) programs have reduced readmissions without impacting emergency center (EC) visits. Our initial pilot OPAT program improved laboratory monitoring without decreasing readmissions or EC visits. We expanded the program and made additive quality improvement interventions.

Outcomes Associated with Outpatient Parenteral Antimicrobial Therapy Interventions in Patients Receiving Care at a Comprehensive Cancer Center

**Figure:**
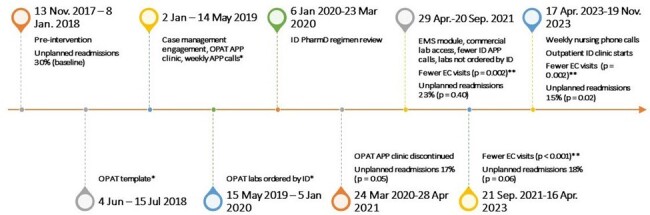

*Previously published initial pilot program. **Compared with baseline among patients with >/= 1 EC visit.

OPAT, Outpatient parenteral antimicrobial therapy; APP, advanced practice provider; EMS, electronic medical system; EC, emergency center; ID, infectious diseases

**Methods:**

We obtained demographic data and baseline frequencies of 30-day unplanned readmissions and EC visits from our electronic medical system (EMS) for 48 patients over 6 weeks prior to pilot implementation. Using the Model for Improvement, we made iterative additions, including pharmacist regimen review, monitoring within the EMS, online commercial laboratory result access, weekly nursing phone calls after discharge, and expansion to infectious disease outpatient starts. After interventions, we compared categorical variables to baseline by using Chi-square or Fisher’s exact test, as appropriate, and compared continuous variables by using nonparametric Wilcoxon rank sum test. We performed multivariable logistic regression to identify risk factors independently associated with readmission.

**Results:**

During June 2018–November 2023, our OPAT program provided care for 1,274 patients. Readmissions decreased from 30% to 15% (p = 0.02) (Figure). Mean number of EC visits per patient decreased (1.3 vs. 1.0, p = 0.002). In multivariable analysis, EMS monitoring and online access to laboratory results were independently associated with decreased readmission risk (aOR 0.22 [95% CI: 0.09-0.56]). Nursing calls after discharge independently protected against readmission (aOR 0.18 [95% CI: 0.07-0.47]). Patients with Eastern Cooperative Oncology Group-Performance Status ≥2 were readmitted more often than those with < 2 (aOR 1.63 [95% CI: 1.05-2.52]).

**Conclusion:**

Improving efficiency and access to laboratory data and weekly nursing phone follow up were associated with fewer readmissions among OPAT patients with cancer. This study is the first, to our knowledge, to show an impact of OPAT on EC visits in any population. OPAT patients with poor functional status have an increased risk for readmission and may benefit from closer monitoring. OPAT programs can improve safety for adult patients with cancer despite competing risks inherent to this population.

**Disclosures:**

Natalie J Dailey Garnes, MD, MPH, MS, AlloVir: Grant/Research Support

